# Establishment of a Lymph Node Metastasis-Associated Prognostic Signature for Lung Adenocarcinoma

**DOI:** 10.1155/2023/6585109

**Published:** 2023-01-31

**Authors:** Jiao Yu, Gang Li, Yingxuan Tian, Shufen Huo

**Affiliations:** ^1^Department of Radiation Oncology, Shaanxi Provincial People's Hospital, Xi'an, Shaanxi 710068, China; ^2^Department of Thyroid and Breast Surgery, Xi'an Daxing Hospital, Xi'an, Shaanxi 710068, China; ^3^Department of Geriatric Respiratory, Shaanxi Provincial People's Hospital, Xi'an, Shaanxi 710068, China

## Abstract

**Background:**

Lung adenocarcinoma (LUAD) is the most common histological subtype of non-small cell lung cancer (NSCLC) with a low 5-year survival rate, which may be associated with the presence of metastatic tumors at the time of diagnosis, especially lymph node metastasis (LNM). This study aimed to construct a LNM-related gene signature for predicting the prognosis of patients with LUAD.

**Methods:**

RNA sequencing data and clinical information of LUAD patients were extracted from The Cancer Genome Atlas (TCGA) and Gene Expression Omnibus (GEO) databases. Samples were divided into metastasis (M) and nonmetastasis (NM) groups based on LNM status. Differentially expressed genes (DEGs) between M and NM groups were screened, and then WGCNA was applied to identify key genes. Furthermore, univariate Cox and LASSO regression analyses were conducted to construct a risk score model, and the predictive performance of model was validated by GSE68465, GSE42127, and GSE50081. The protein and mRNA expression level of LNM-associated genes were detected by human protein atlas (HPA) and GSE68465.

**Results:**

A prognostic model based on eight LNM-related genes (ANGPTL4, BARX2, GPR98, KRT6A, PTPRH, RGS20, TCN1, and TNS4) was developed. Patients in the high-risk group had poorer overall survival than those in the low-risk group, and validation analysis showed that this model had potential predictive value for patients with LUAD. HPA analysis supported the upregulation of ANGPTL4, KRT6A, BARX2, RGS20 and the downregulation of GPR98 in LUAD compared with normal tissues.

**Conclusion:**

Our results indicated that the eight LNM-related genes signature had potential value in the prognosis of patients with LUAD, which may have important practical implications.

## 1. Background

Almost a quarter of cancer-related deaths are caused by lung cancer, which ranks among the top ten causes of cancer deaths in both men and women [1]. Non-small-cell lung cancer (NSCLC) accounts for approximately 85% of lung cancer cases, of which lung adenocarcinoma (LUAD) is the most common histological subtype, accounting for 60% of cases [2]. With the improvement of treatment approaches, the mortality of LUAD has been declining steadily year by year. However, the 5-year survival rate remains still low. Studies have indicated that the poor prognosis of most LUAD patients is due to the presence of metastatic tumors at the time of diagnosis [3, 4]. Notably, lymph node metastasis (LNM) is the most common form [5]. Thus, there is an urgent need to elucidate the molecular mechanisms of LNM in LUAD.

The lymphatic system is the main route of LUAD metastasis, and lymphatic metastasis is an important indicator influencing the prognosis and staging of it [6]. It has been reported that the 5-year survival rate of LUAD patients with LNM is only 27%, while the 5-year survival rate of those without LNM is more than 95% [7]. Therefore, identifying specific biomarkers of LNM is helpful for the diagnosis and treatment of LUAD. Previous studies have screened several genes related to LUAD metastasis. For example, Jiang et al. [8] revealed that PTK7 served a carcinogenic role in LUAD and might be a molecular biomarker of LNM; Zhang et al. [9] indicated that overexpression of Rab27b was associated with the malignant properties of LUAD, and it might be considered as a potential indicator of LNM and prognosis. Despite these encouraging findings, the clinical impact of a single gene is limited. In recent years, with the development of large-scale genome analysis techniques, numerous studies have demonstrated gene signatures for survival prediction and risk stratification of LUAD patients [10]. The published models are constructed mainly based on the immune or autophagy-associated genes [11–13]; however, few studies have proposed LNM-related prognostic models to predict the overall survival of patients with LUAD.

In this study, we explored the potential prognostic value of LNM genes in LUAD via integration of the LNM-associated genes and clinical data obtained from The Cancer Genome Altas (TCGA) portal. Samples from the TCGA database were divided into metastasis (M) and nonmetastasis (NM) groups, and then genes differentially expressed in these two groups were identified. Next, weighed gene coexpression network analysis (WGCNA) was performed to screen the key modules and genes related to LNM, followed by LASSO regression analysis to construct an optimal prognostic model. Further, the predictive performance of model was assessed by using three gene expression omnibus (GEO) datasets. Meanwhile, the expression level of genes in the model was assessed by using the GEO dataset and human protein atlas (HPA) database. The constructed model could be used as a prognostic signature to improve the management of metastatic patients, which might ultimately be applied to assist clinicians in treatment selection and prognostic evaluation for LUAD patients with LNM.

## 2. Methods

### 2.1. Data Collection and Preprocessing

The mRNA expression data and clinical follow-up information of 505 LUAD samples were downloaded from the TCGA database (2021/09/10 analysis archive; https://gdc-portal.nci.nih.gov/). Among these, the N0 stage was considered as the NM group and N1–N3 stages were regarded as the M group. Next, after eliminating the samples with missing information on survival time, 493 samples remained to construct the prognostic model.

Moreover, three external datasets downloaded from the GEO database (https://www.ncbi.nlm.nih.gov/) were employed as validation cohorts, including GSE68465 (based on the GPL96 platform) [14], GSE42127 (based on the GPL6884) [15, 16], and GSE50081 (based on the GPL570) [17]. After eliminating the patients without complete survival data, 700 samples were included in further analyses: 442 in GSE68465, 131 in GSE42127, and 127 in GSE50081. Similarly, these samples were divided into NM and M groups according to the metastatic state.

### 2.2. Screening of Differentially Expressed Genes (DEGs) between M and NM Groups

Differentially expressed analysis between M and NM groups was conducted by using the limma package of R software (Version 3.6.1; https://bioconductor.org/packages/release/bioc/html/limma.html) [18], and genes with *p* value < 0.05 and |log_2_ fold change (FC)| > 0.5 were considered as DEGs. The heatmap for DEGs was plotted via pheatmap package (Version 1.0.8; https://cran.r-project.org/web/packages/pheatmap/index.html) and the volcano plot was visualized by the ggplot2 package in R. Furthermore, DAVID (Version 6.8; https://david.ncifcrf.gov/) was employed to perform Gene ontology (GO) enrichment and Kyoto Encyclopedia of Genes and Genomes (KEGG) pathway analyses. The *p* value less than 0.05 as the threshold for significant enriched terms.

### 2.3. Screening of Disease-Related Modules and Genes Using WGCNA

The WGCNA approach is used to excavate the gene modules that are highly related to the sample phenotype in high-throughput data; among these, the most central genes are identified as key genes that serve a crucial role in the module. In this analysis, with the lymph node metastasis and nonmetastasis as properties, the WGCNA package (Version 1.61, https://cran.r-project.org/web/packages/WGCNA/) [19] was used to analyze the entire gene of TCGA-LUAD. The top 75% of the genes with the median absolute deviation (MAD) were selected, and then MAD value > 0.01 were extracted to conduct the WGCNA algorithm. Next, Venn analysis was used to screen the overlapping genes between DEGs and genes in modules for subsequent analysis.

### 2.4. Establishment of a Prognostic Model

Further, 493 samples with complete prognostic information in the TCGA database were used as a training cohort to develop the prognostic model. Based on the mRNA expression levels of the overlapping genes, univariate Cox regression analysis was performed by using the survival package (Version 2.41-1; https://bioconductor.org/packages/survivalr/) to screen prognosis-related genes, with *p* value < 0.05 as cutoff value. Then, optimal gene signature was obtained via LASSO analysis using Lars package (Version 1.2; https://cran.r-project.org/web/packages/lars/index.html). The following formula was used to calculate the risk score (RS): RS = ∑Coef_gene_ × Exp_gene_. Here, Coef represents the LASSO coefficient, and Exp represents the expression level of the gene.

### 2.5. Performance Assessment of the RS Model

After calculating the RS, patients in the TCGA, GSE68465, GSE42127, and GSE50081 datasets were divided into high-risk (HR) and low-risk (LR) groups based on the median value of RS. The Kaplan–Meier (KM) approach was used to evaluate the association between the different risk groups and LUAD prognosis. In addition, the receiver operating characteristic (ROC) curves were plotted to evaluate the prognostic performance of the RS model. The area under the ROC curve (AUC) at different endpoints (1, 3, and 5 years) was calculated by using the time ROC package (Version 0.4) in R3.6.1.

### 2.6. Gene Set Enrichment Analysis (GSEA) of the HR and LR Groups

GSEA was used to analyze the functional pathways enriched by HS and LS groups, and nominal (NOM)*p* value < 0.05 and ∣normalized enrichment score (NES)∣ > 1 were set as cutoff threshold criteria.

### 2.7. Prognostic Characteristics of Genes in the RS Model

In the training cohort, the KM method was used to compare the survival time of each gene between the HR and LR groups by using the survival package (Version 2.41-1).

### 2.8. Correlation Analysis of RS and Clinical Characteristics

By combining the clinical information from LUAD, the correlation between RS and clinical characteristics (age, gender, T, N, and stage) was analyzed. *p* value < 0.05 was considered statistically significant.

### 2.9. HPA Validation

The immunohistochemical staining map of genes in the RS model was downloaded from the HPA database (https://www.proteinatlas.org/) to verify the difference in protein expression levels of genes between normal and tumor groups.

### 2.10. Methylation Analysis

The correlation between each biomarker and its corresponding methylation site as well as copy number was analyzed using MEXPREWSS (https://mexpress.be/).

### 2.11. Gene Expression Validation

Next, we used an independent dataset (GSE68465) to verify the mRNA expression levels of genes in the RS model. The paired *t*-test in R was applied to validate the difference in expression level of biomarkers between the M and NM groups.

## 3. Results

### 3.1. Screening of DEGs and Functional Enrichment Analysis

**Figure 1:**
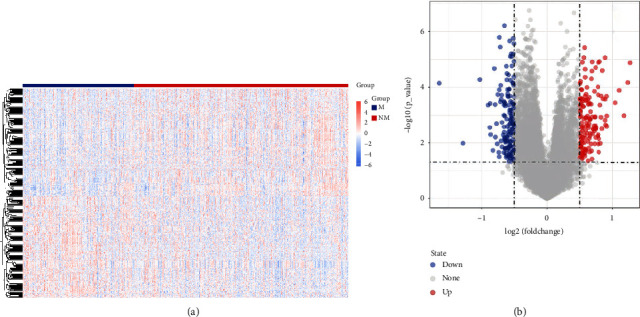
Identification of differentially expressed genes (DEGs) between metastasis (M) and nonmetastasis (NM) groups. (a) Heatmap of DEGs between M and NM groups. Blue box indicates the M group and red box indicates NM group. (b) Volcano plot showing the DEGs between M and NM groups in TCGA cohort. Blue node represents a lower expression of gene in the M group, and red node represents a higher expression of gene in the M group.

According to the state of cancer metastasis, 172 and 333 samples in the TCGA database were classified into NM and M groups, respectively. A total of 294 DEGs were screened between M and NM groups. The specific distribution of DEGs was visualized by the heatmap (Figure 1(a)) and volcano plot (Figure 1(b)).

**Figure 2:**
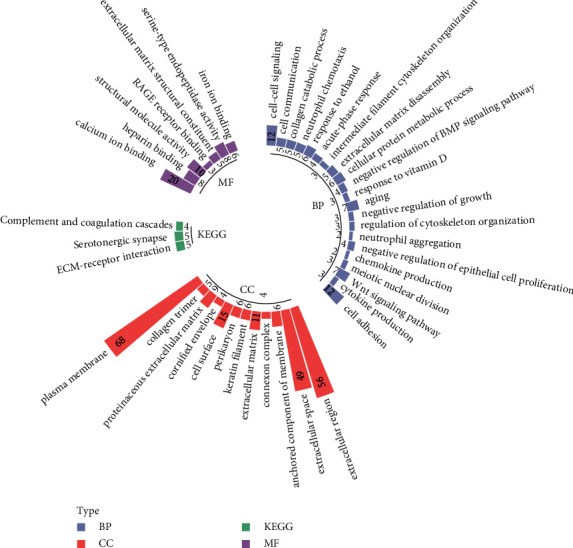
Functional enrichment analysis of DEGs. Lilac stands for gene ontology (GO)_biological processes (BP) category, orange stands for GO_cellular component (CC) category, modena stands for GO_molecular function (MF) category, and green stands for Kyoto Encyclopedia of Genes and Genomes (KEGG) pathway. The number in the box represents the count of genes enriched in the term.

Functional enrichment analyses showed that these DEGs were significantly enriched in 21 GO-biological process (BP) terms, 12 GO-cellular component (CC) terms, 7 GO-molecular function (MF) terms, and 3 KEGG pathways (Figure 2). In brief, DEGs were mainly enriched in GO-BP terms such as cell-cell signaling and cell adhesion; enriched in GO-CC terms such as collagen trimer and extracellular region; and enriched in GO-MF terms such as calcium ion binding and structural molecule activity. In terms of KEGG pathways, DEGs were involved in ECM-receptor interaction, serotonergic synapse, and complement and coagulation cascades.

### 3.2. Identification of LUAD-Related Hub Modules and Genes

**Figure 3:**
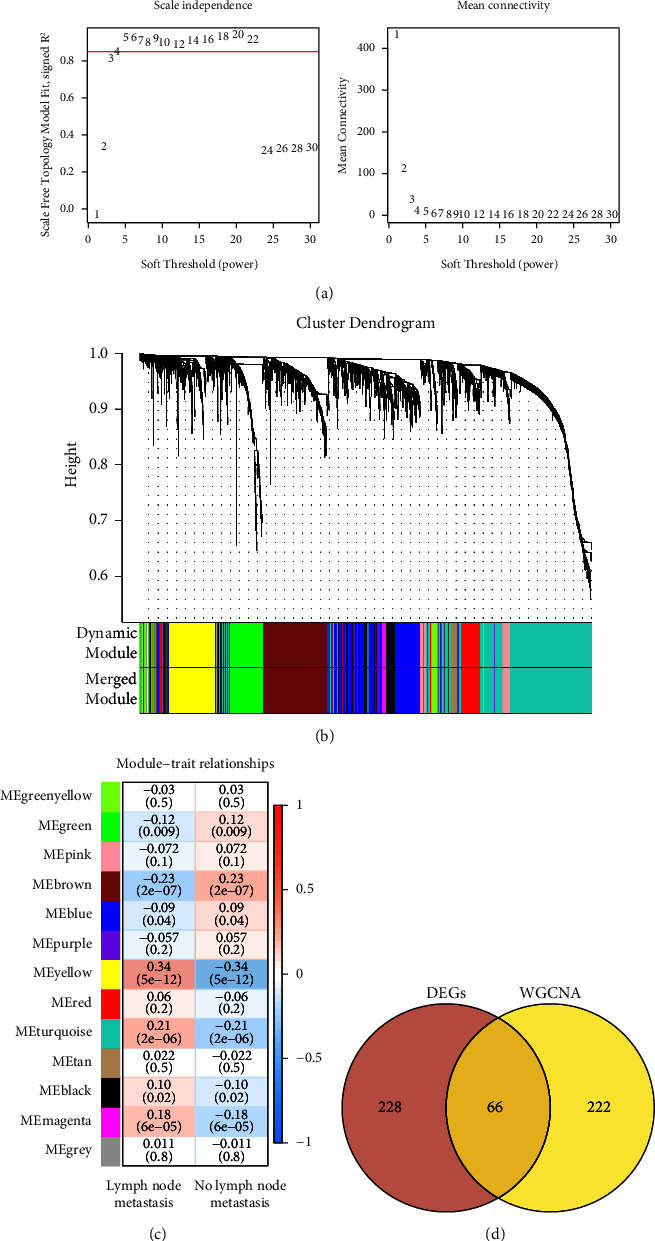
Results of weighted gene coexpression network analysis. (a) Left panel shows adjacency matrix weight parameter power selection plot. *X* axis represents the power value and *Y* axis represents the square of the correlation coefficient between log (*k*) and log (*p* (*k*)) in the corresponding network. Horizontal red line: 0.85. Right panel shows the mean connectivity (*Y*-axis) as a function of the soft-thresholding power (*Y*-axis). (b) Cluster dendrogram of the coexpression network modules. Each color represents a different module. (c) Association between the gene modules and metastasis state. The left column is lymph node metastasis and right column is no lymph node metastasis. (d) Venn diagram of the intersection of DEGs and yellow module genes.

As shown in Figure 3(a), we selected the value of power when the scale-free*R*^2^ reached to 0.85 for the first time (red line), that is, power = 4. Based on the hierarchical clustering and dynamic tree-cutting algorithms, highly correlated genes were clustered into modules, and finally 13 modules were obtained (Figure 3(b)). Next, the correlation between each module and LNM was assessed. Results indicated that four modules including yellow (*r* = 0.34, *p* = 5*E* − 12), turquoise (*r* = 0.21, *p* = 2*E* − 06), black (*r* = 0.10, *p* = 0.02), and magenta (*r* = 0.18, *p* = 6*E* − 05) were positively correlated with the LNM (Figure 3(c)). Among these, the yellow module had the highest correlation with LNM, which was regarded as the metastasis-related significant module for further analysis. In this module, 288 LNM-related genes were contained, and then genes were integrated with the above DEGs. In total, 66 overlapping genes were obtained for subsequent analyses (Figure 3(d)).

### 3.3. Construction of the RS Model Based on Overlapping Genes

**Figure 4:**
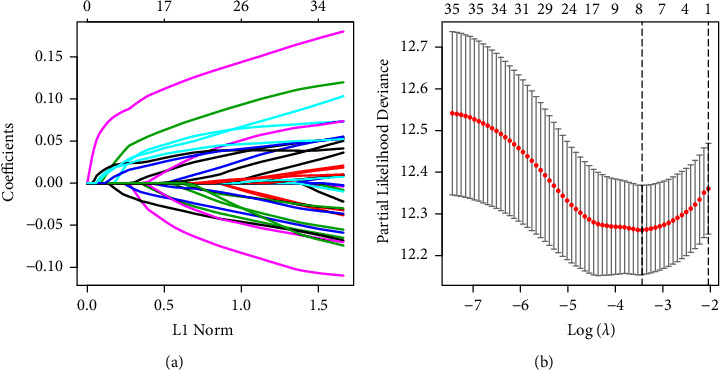
Identification of prognostic signature in the TCGA cohort. (a) Calculation of LASSO coefficient for each lambda. Each line represents a gene confidence value. (b) Partial likelihood deviance of LASSO coefficient. The two vertical dashed lines represent lambda.min (left line) and lambda.1se (right line). Horizontal axis represents the log (*λ*) value, while vertical axis represents the partial likelihood deviance of the log (*λ*) value.

First, univariate Cox regression analysis showed 36 genes had prognostic values. Next, LASSO analysis indicated that eight was considered as the optimal number based on the lambda values (Figures 4(a) and 4(b)). Eight genes were ANGPTL4, BARX2, GPR98, KRT6A, PTPRH, RGS20, TCN1, and TNS4. According to the expression level and LASSO coefficient of each gene, the RS model was constructed using the following formula: RS = (0.0365 *∗* Exp_ANGPTL4_) + (0.0158 *∗* Exp_BARX2_) + (−0.0131 *∗* Exp_GPR98_) + (0.0232 *∗* Exp_KRT6A_) + (0.024 *∗* Exp_PTPRH_) + (0.0852 *∗* Exp_RGS20_) + (0.0124 *∗* Exp_TCN1_) + (0.0211 *∗* Exp_TNS4_).

### 3.4. Validation of Predictive Performance for the RS Model

**Figure 5:**
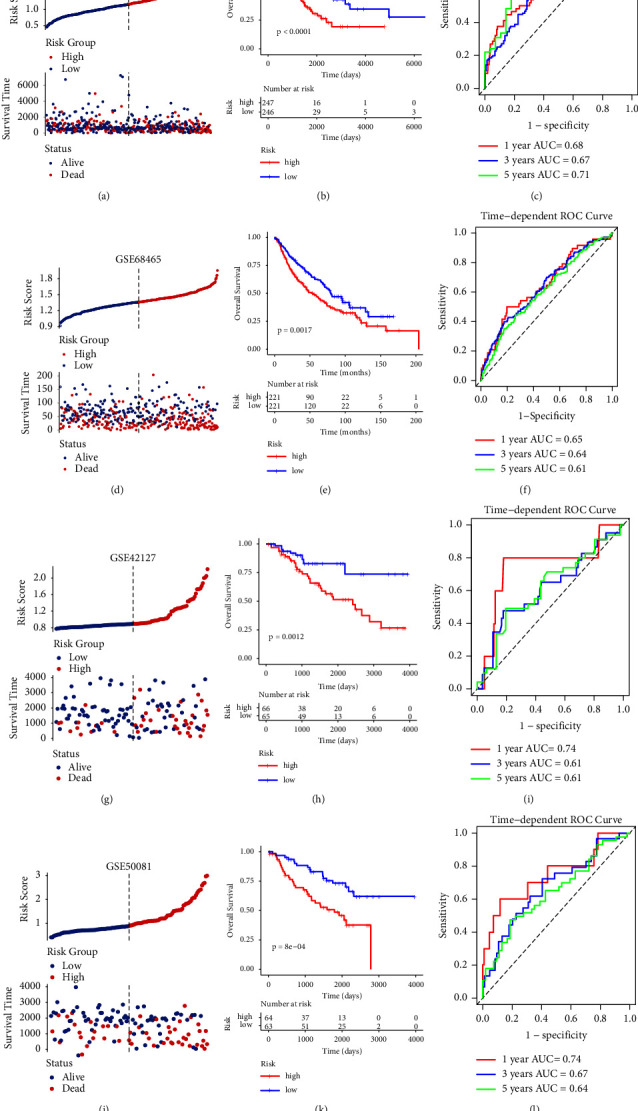
Construction and validation of metastasis-associated signature model in the training and three validation cohorts. (a) The distribution and survival state of samples in the TCGA cohort. (b) Kaplan–Meier (KM) survival curves for samples in the high-risk (HR) and low-risk (LR) groups of TCGA cohort. (c) Receiver operating characteristic (ROC) analysis of overall survival at 1, 3, and 5 years in TCGA cohort. (d) The distribution and survival state of samples in the GSE68465. (e) KM survival curves showing survival outcomes of GSE68465. (f) ROC analysis of GSE68465. (g) Distribution of patients in GSE42127 based on the median RS and survival status for each case. (h) Overall survival curves for patients in LR and HR groups in GSE42127 dataset. (i) ROC curve showed the predictive efficiency of the RS in GSE42127. (j) RS distribution and survival status distribution of patients in GSE50081. (k) KM curves of overall survival in GSE50081. (l) ROC curves for 1-, 3-, and 5-year survival in GSE50081.

In the training and validation sets, samples were assigned into HR and LR groups based on the median of RS. In the TCGA training set, the distribution and survival status of patients are presented in Figure 5(a). The patients in the LR group had significantly shorter overall survival than those in the HR group (Figure 5(b)). The AUC of ROC curves for 1, 3, and 5 years were 0.68, 0.67, and 0.71, respectively, indicating that the RS model had good accuracy and specificity (Figure 5(c)). Moreover, these findings were confirmed by the validation datasets. In brief, in the GSE68465, patients in the HR group had more dead cases (Figure 5(d)) and had a poor survival time (Figure 5(e)). The ROC curve indicated that AUC was 0.65, 0.64, and 0.61 at 1, 3, and 5 years (Figure 5(f)). In the GSE42127, more alive cases were observed in the LR group (Figure 5(g)). The KM curve indicated that patients in the HR group showed a significantly lower probability of survival compared to the LR group (*p* < 0.05, Figure 5(h)). The ROC analysis revealed that AUC values for 1-, 3-, and 5-year OS were 0.74, 0.61, and 0.61, respectively (Figure 5(i)). In the GSE50081, patients with higher RS were more likely to have a poor prognosis (Figure 5(j)). Meanwhile, survival curves showed that overall survival was significantly lower in the HR group than in the LR group (*p* < 0.05, Figure 5(k)). Results of the AUC for 1-, 3-, and 5-year OS were 0.74, 0.67, and 0.64, respectively (Figure 5(l)). Altogether, these data suggested that the predictive performance of the model was superior.

### 3.5. Different Pathway in HR and LR Groups Analyzed by GSEA

**Figure 6:**
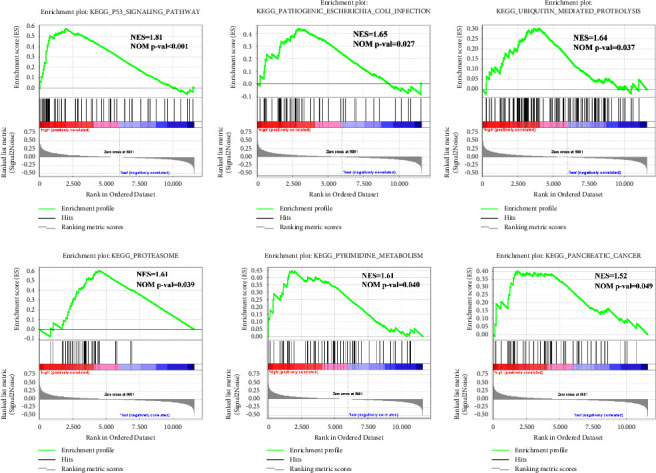
Functional enrichment analysis based on the RS model by GSEA. Six significantly enriched KEGG pathways in the HR group.

Fourteen different signaling pathways were identified between the HR and LR groups. Among these, eight pathways were associated with the LR group, such as valine leucine and isoleucine degradation, taste transduction, and nitrogen metabolism; six pathways were closely corrected with the HR group, including the P53 signaling pathway, pathogenic Escherichia coli infection, ubiquitin-mediated proteolysis, the proteasome, pyrimidine metabolism, and pancreatic cancer (Figure 6).

### 3.6. Prognostic Value Analysis of Each Gene in the RS Model

**Figure 7:**
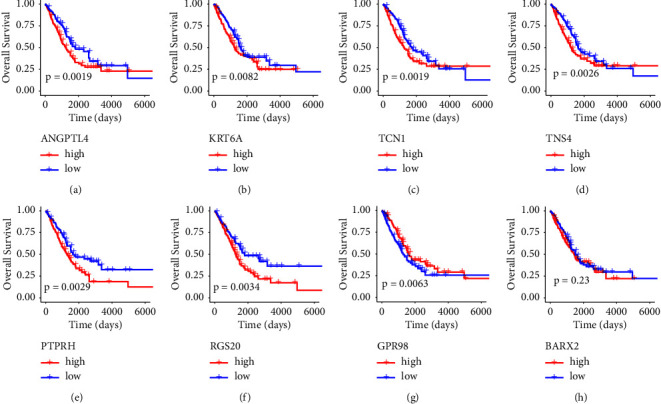
KM survival analysis of patients divided into the low- and high-expression groups of eight signature genes in the TCGA cohort. (a) ANGPTL4; (b) KRT6A; (c) TCN1; (d) TNS4; (e) PTPRH; (f) RGS20; (g) GPR98; (h) BARX2. Blue line represents low-expression group and red line represents high-expression group.

Based on the median of gene expression level, patients were divided into low-expression and high-expression groups. KM curves showed that patients with low expression of ANGPTL4, KRT6A, TCN1, TNS4, PTPRH, and RGS20 had significantly longer survival times (*p* value < 0.05, Figures 7(a)–7(f)); a high-expression level of GPR98 was associated with longer overall survival (*p* value < 0.05, Figure 7(g)). Although there was no significant difference, we observed that high gene expression of BARX2 was connected with a poor prognosis (Figure 7(h)).

### 3.7. Correlation Analysis of Gene Signatures and Clinical Features

**Figure 8:**
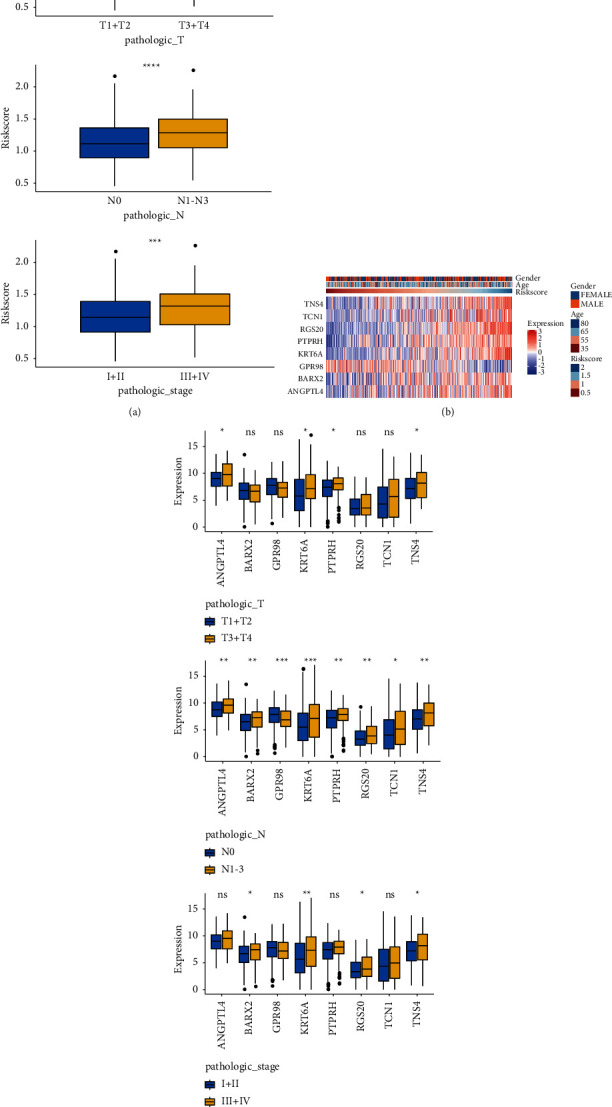
Correlation analysis of RS and clinical characteristics. (a) RS in subgroups of the T stages (T1 + T2 vs. T3 + T4), N stages (N0 vs. N1–N3), or pathological stages (I + II vs. III + IV). (b) Heatmaps showing the mRNA expression of eight selected genes in TCGA cohort. (c) Correlation of each gene and clinical characteristics (T stages, N stages, and pathological stages). Ns, no significance; ^∗^*p* value < 0.05; ^∗∗^*p* value < 0.01; ^∗∗∗^*p* value < 0.001; ^∗∗∗∗^*p* value < 0.0001.

The correlation analysis revealed that patients with higher RS were significantly with higher T stage (T3 + T4), higher N stage (N1–N3), and advanced stages (stage III + IV) (Figure 8(a)). A heatmap showed that genes included TNS4, TCN1, RGS20, PTPRH, KRT6A, BARX2, and ANGPTL4 were up-regulated in the HR group, while *GPR98* was down-regulated in the HR group (Figure 8(b)). Meanwhile, the relationship between each gene and clinical features was calculated, and results showed that KRT6A and TNS4 were significantly associated with these three indicators (Figure 8(c)).

### 3.8. Immunohistochemical Verification of Genes Using HPA

**Figure 9:**
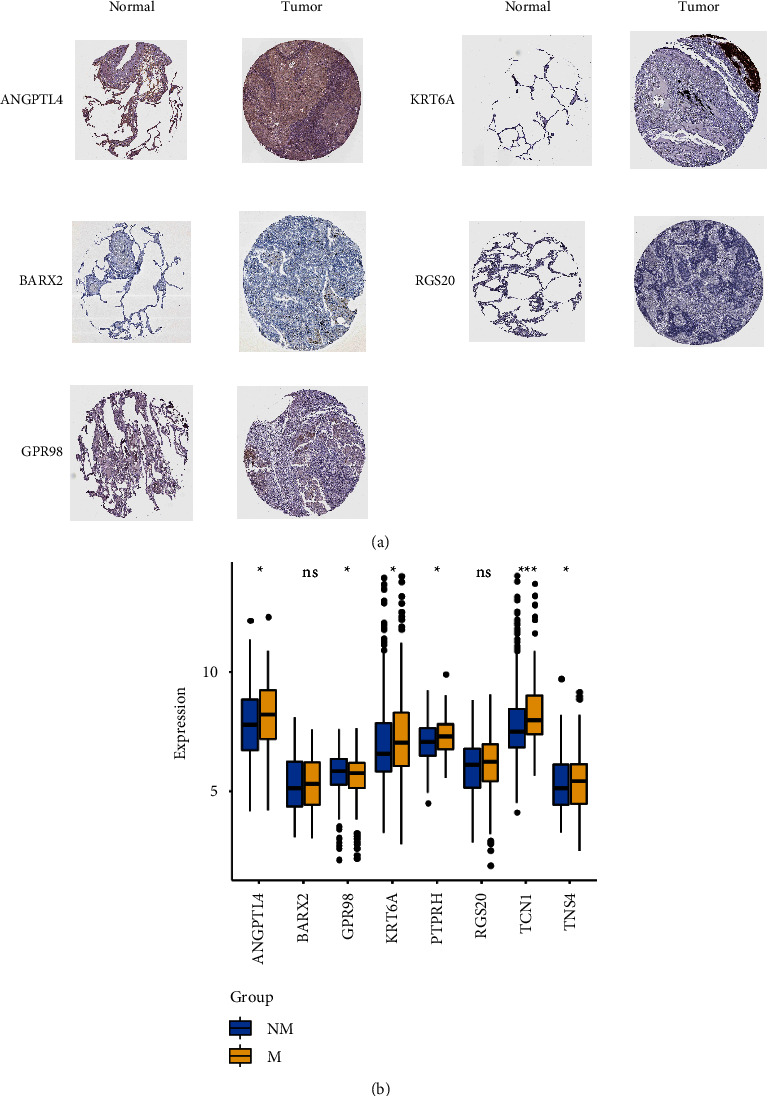
Validation of the expression level of genes in the RS model. (a) Protein expression level of five genes in LUAD and normal tissues based on immunohistochemistry results from the human protein atlas (HPA) database. (b) mRNA expression level of RS model genes in M and NM groups in GSE68465. Blue indicates NM sample and yellow indicates M sample.

The HPA database was applied to display the protein level of genes in the RS model. The immunohistochemical images of PTPRH, TCN1, and TNS4 were not recorded in this database. The representational plots of ANGPTL4, KRT6A, BARX2, RGS20, and GPR98 are shown in Figure 9(a). Compared with the normal samples, the protein expression levels of ANGPTL4, KRT6A, BARX2, and RGS20 were higher, while the expression level of GPR98 was lower in the LUAD samples, which was consistent with the above findings.

### 3.9. Methylation Analysis of Biomarkers

The methylation sites and copy number of genes in the RS model were analyzed by using the MEXPREWSS website, while the information for GPR98 was not retrieved in this database. We found that ANGPTL4, BARX2, KRT6A, PTPRH, RGS20, TCN1, and TNS4 were significantly associated with 8, 12, 6, 15, 19, 3, and 8 methylation sites, respectively.

### 3.10. Validation of mRNA Expression Levels of Genes

To further observe the expression level of genes, we used GSE68465 to verify the difference in the mRNA expression level between the M and NM groups. As shown in Figure 9(b), ANGPTL4, KRT6A, PTPRH, TCN1, and TNS4 were significantly higher in the M group than those in the NM group; while the expression level of GPR98 was markedly decreased in the M group (all *p* value < 0.05).

## 4. Discussion

LNM is one of the main factors affecting the prognosis of LUAD, and it significantly reduces the survival rate of patients with LUAD [20], which is considered as an important predictor of poor prognosis. Therefore, credible prognostic signatures related to LNM status may provide a great prospect for identifying potential therapeutic targets and enhancing patient management. In this study, an eight LNM-related genes model, including ANGPTL4, BARX2, GPR98, KRT6A, PTPRH, RGS20, TCN1, and TNS4, was developed. Our RS model could effectively stratify patient outcomes in the LUAD and was validated in GSE68465, GSE42127, and GSE50081. Based on the median of RS, patients in TCGA and GEO were divided into HR and LR groups, and patients in the HR group had a poor prognosis. These findings meant that our bioinformatics analysis using TCGA and GEO cohorts had prognostic value, and the identified genes might serve as potential markers for LUAD.

The focus of this study was to compare M with NM samples, and the screened DEGs were associated with LNM states. Eight key genes were further obtained via univariate and LASSO regression analyses. Angiopoietin-like 4 (ANGPTL4) encodes a glycosylated secreted protein that acts as a serum hormone to regulate blood glucose homeostasis and lipid metabolism; meanwhile, the encoded protein can serve as an apoptotic survival factor for vascular endothelia cells that may prevent metastasis by inhibiting vascular growth and tumor cell invasion [21, 22]. Previous studies confirmed that ANGPTL4 was significantly associated with vein invasion and tumor invasion depth in human colorectal cancer, and all patients with distant metastases presented immunopositive for ANGPTL4, suggesting that ANGPTL4 could promote distant metastasis [23]. Moreover, Mo et al. [24] established a nine-gene signature that was observably connected with metastasis and prognosis of LUAD patients, of which ANGPTL4 was also contained. BARX homeobox 2 (BARX2) encodes a member of the homeobox transcription factor family, which controls cell adhesion and actin cytoskeleton remodeling [25]. Evidence has indicated that it may be a molecular switch that controls cell differentiation and proliferation [26]. BARX2 was enriched in the epithelial-mesenchymal transition (EMT) pathway, and it was involved in tumorigenesis and the development of LUAD [27]. GPR98, also called Adhesion G protein-coupled receptor V1 (VLGR1), encodes a member of the G protein-coupled receptor superfamily. Previous study showed that there were 30 alternative exon usage of GPR98 significantly associated with survival of glioblastoma multiforme [28]. Keratin 6A (KRT6A) encodes a family member of type II cytokeratins, which is involved in the EMT pathway. Yang et al. [29] observed that KRT6A was up-regulated in LUAD tissues and overexpression of it was associated with poor prognosis; meanwhile, KRT6A promoted migration and proliferation of lung cancer cells, indicating that KRT6A could be used as a prognostic biomarker for LUAD. The protein encoded by protein tyrosine phosphatase receptor type H (PTPRH) belongs to the protein tyrosine phosphatase (PTP) family that regulates a variety of cellular processes, such as cell growth, differentiation, and oncogenic transformation [30]. Existing studies have reported the relationship between PTPRH and LUAD. For example, Chen et al. [31] observed that PTPRH was overexpressed in the LUAD tissue and served as an independent prognostic factor for LUAD. Previous studies indicated the prognostic value of regulator of G protein signaling 20 (RGS20) in patients with LUAD, and it might be a novel prognostic marker for LUAD [32]. Meanwhile, the expression level of RGS20 was elevated in metastatic cancer cells, and then the migration and invasion abilities of NSCLC cell lines (A549 and H1299) were impaired when RGS20 was stably knocked out, suggesting that RGS20 might accelerate the metastasis of tumor cells [33]. Transcobalamin 1 (TCN1) encodes a member of the vitamin B12-binding protein family, and it plays multiple roles in maintaining the basic functions of cell proliferation and metabolism [34]. TCN1 acts as a biomarker for the prognosis of various cancers, including colon cancer [35], gastric cancer [36], and LUAD [37], and it could promote the migration and invasion of cancer cells. Tensin 4 (TNS4) is a protein coding gene that promotes cell movement through GPCR signal transduction and the EMT pathways [38]. Furthermore, TNS4 was associated with the prognosis of LUAD [39], and it served an important role in the migration and invasion of gastric cancer [40]. Taken together, these studies emphasized that the identified genes were involved in cancer progression and could serve as prognostic markers for LUAD. Nevertheless, the relationship between GPR98 and metastasis of LUAD has not been reported, which requires further investigated in clinical experiments.

After constructing the RS model, the patients were divided into LR and HR groups. Survival analysis revealed that patients in the HR group had shorter overall survival than those in the LR group. Next, performance evaluation showed that the established model had better performance in predicting the prognosis of LUAD patients. To explore the pathways involved in the gene sets from the HR group, a GSEA was performed. Results revealed that several key pathways were closely corrected with the HR group, such as P53 signaling pathway, pathogenic escherichia coli infection, and ubiquitin-mediated proteolysis. Evidence indicated that genes such as PAQR3 could regulate the progression of NSCLC via the p53 signaling pathway [41], and genes involved in this pathway may play roles in distant metastasis and LNM [42]. Luo et al. [43] revealed that the TUBB gene was enriched in pathogenic Escherichia coli infection, which was associated with the progression of pancreatic cancer. A defect in genes participating in the ubiquitin-mediated proteolytic pathway could cause a series of human diseases, such as cancer [44]. Thus, we speculated that genes might influence the status of LNM by regulating these pathways, which further affected the prognosis of LUAD.

Based on the gene expression profiling from public databases (TCGA and GEO) and a two-step design including development and validation, our study provided reliable evidence for the value of the LNM-related gene signature in the prognostic evaluation of LUAD. Although there have been studies to construct a prognostic model of LUAC based on LNM-related genes [45], the advantage of our research was to select genes significantly related to LNM via WGCNA for further analysis. However, some limitations should be noted. The sample size of this analysis was small, and it was necessary to verify the predictive accuracy of the model in a large-scale clinical sample. In addition, the biological function and specific mechanism of these identified genes in LNM of LUAD were still unclear, so we will elucidate their contents in the future work.

## 5. Conclusion

In summary, we constructed and validated a LNM-related gene signature to predict the prognosis for patients with LUAD. This prognostic model contained eight genes and had better specificity and predictive performance, which may assist clinicians in making a correct diagnosis and discovering the prognostic risk of LUAD patients in advance.

## Data Availability

The datasets used and/or analyzed during the current study are available from the corresponding author on reasonable request.

## Abbreviations

LUAD: Lung adenocarcinoma
LNM: Lymph node metastasis
TCGA: The Cancer Genome Atlas
GEO: Gene expression omnibus
M: Metastasis
NM: Nonmetastasis
DEGs: Differentially expressed genes
NSCLC: Non-small-cell lung cancer
WGCNA: Weighed gene coexpression network analysis
GO: Gene ontology
KEGG: Kyoto Encyclopedia of Genes and Genomes
MAD: Median absolute deviation
LR: Low risk
KM: Kaplan–Meier
ROC: Receiver operating characteristic
HPA: Human protein atlas
ANGPTL4: Angiopoietin like 4
BARX2: BARX homeobox 2
EMT: Epithelial-mesenchymal transition
KRT6A: Keratin 6A
PTPRH: Protein tyrosine phosphatase receptor type H
PTP: Protein tyrosine phosphatase
RGS20: Regulator of G protein signaling 20
TCN1: Transcobalamin 1
TNS4: Tensin 4.
